# Modeling the relationship between gene expression and mutational signature

**DOI:** 10.15302/J-QB-022-0309

**Published:** 2023-01-13

**Authors:** Limin Jiang, Hui Yu, Yan Guo

**Affiliations:** Department of Internal Medicine, Comprehensive Cancer Center, University of New Mexico Albuquerque, NM 87109, USA

**Keywords:** mutational signature, gene expression, support vector machine, random forest, extreme gradient boost

## Abstract

**Background:**

Mutational signatures computed from somatic mutations, allow an in-depth understanding of tumorigenesis and may illuminate early prevention strategies. Many studies have shown the regulation effects between somatic mutation and gene expression dysregulation.

**Methods:**

We hypothesized that there are potential associations between mutational signature and gene expression. We capitalized upon RNA-seq data to model 49 established mutational signatures in 33 cancer types. Both accuracy and area under the curve were used as performance measures in five-fold cross-validation.

**Results:**

A total of 475 models using unconstrained genes, and 112 models using protein-coding genes were selected for future inference purposes. An independent gene expression dataset on lung cancer smoking status was used for validation which achieved over 80% for both accuracy and area under the curve.

**Conclusion:**

These results demonstrate that the associations between gene expression and somatic mutations can translate into the associations between gene expression and mutational signatures.

## Author summary

To overcome the limitations of non-negative matrix factorization in the situation of sparse mutation, a method was designed in this paper to predict mutational signatures based on RNA-seq data. This method first was used to build the associations between gene expression and 49 established mutational signatures. Then, a total of 587 successful models covering 31 cancer types were obtained based on the condition of accuracies and AUCs (Area-Under-Curve) are both greater than 0.8. Finally, all successful models were assembled to form an online tool (EMSI) as a component of the MutEx analysis suite, and models can be visited at the website of innovebioinfo.

## INTRODUCTION

Somatic mutation is considered the primary direct cause of tumorigenesis. A cancer patient can carry up to tens of thousands of single base substitution somatic mutations. Accumulating throughout a lifespan, somatic mutations are ascribed to multiple mutational causes, such as erroneous DNA replication/DNA repair processes, endogenous/exogenous mutagen exposures, and enzymatic modification of DNA. Endogenous mutagens such as reactive oxygen species are generated within the organism; exogenous mutagens are environmental sources outside the affected organism. There are three major types of exogenous mutagens: physical, chemical, and biological. Physical mutagens mainly consist of different forms of radiation, especially ultraviolet (UV) light. Examples of well-known chemical mutagens are aromatic amines, commonly found in tobacco. Biological mutagens comprise primarily pathogenic viruses, such as human papilloma virus, and hepatitis virus.

Somatic mutations occur as the consequence of a mutational process that is triggered by either endogenous errors or exogenous mutagens. At a particular chromosome locus, the somatic mutational process induces a new nucleotide allele that is different from the intrinsic allele(s) in the nuclear genome. Speaking of single nucleotide substitutions only, such mutational processes always result in non-hereditary alterations between the four types of nucleotides: adenine (A), guanine (G), cytosine (C), thymine (T). Taking into consideration DNA’s complementarity, six distinct types of substitutions can be formed between the four nucleotides. When adding up the 5′-neighbor and the 3′-neighbor, a three-nucleotide motif can be derived from the focal substitution, and thus expand the six-substitution inventory to a 96-motif catalog [1]. The profile of various mutational motifs in a cancer patient can be modeled as a combination of varied contributions from distinct mutational signatures. A mutational signature is conceived as the footprint of a mutational process in the nuclear genome, represented in the form of relative frequencies of the motifs of a mutational catalog. The concept of mutational signatures was originally introduced in 2013 [2] and validated with cancer cell lines [3]. Since then, a series of high profile studies [4–7] was conducted to explore the association of mutational signatures with cancers. Mutational signature has been hailed as a powerful vehicle for pinpointing the role of critical environmental exposures in cancer development. The sequencing of human cancer genomes has revealed a multitude of mutational signatures that point to causal exposures. For example, skin cancers associated with UV exposure have a preponderance of C>T mutations, consistent with the pattern of known UV dependent photodimers [8]. Characteristic mutational signatures have been identified for exposures to tobacco and plant toxins (*e.g*., aflatoxin) [9]. Moreover, as mutational signatures associated with the infidelity of DNA repair process are being discovered, the molecular mechanisms underlying DNA repair became better understood. Additionally, longitudinal mutation accumulation is attributed to certain mutational processes with clock-like properties correlated with age/time that can be identified by distinct mutational signatures [10]. In a recent study, a compendium of mutational signatures resulting from environmental agents (arsenic, UV light, *etc*.) was compiled [11].

Current methods utilize the frequency of 96-motif catalog to predict mutational signatures, and these computational methods mostly comprise non-negative matrix factorization and least square linear regression. Because the performance of current computational methods is suboptimal in case of sparse mutations [12], it is necessary to develop new methods to uncover mutational signatures in a novel perspective. Fortunately, gene expression patterns are often characterized for cancer-related genes to inform cancer diagnosis or therapeutics. Aberrant gene expression can be caused by somatic mutations or the consequential disruption of normal gene regulation. An early study has shown that somatic mutations caused dysregulation of gene expression of multiple glioblastoma multiforme-related genes [13]. Recently, a study suggested the associations between somatic mutation and gene expression alteration can be capitalized upon to determine somatic mutation impact [14]. However, integrative study combining somatic mutation and gene expression has been sporadic. The concept of modeling the regulatory effect between somatic mutation and gene expression is similar to the concept of gene expression quantitative trait loci (eQTL), which models gene expression by germline variations. Based on these aforementioned studies and concepts, we set out to model mutational signatures based on gene expression. We found that a portion of the reference mutational signatures are correlated with gene expression. In this paper, our novel models are developed based on gene expression, which resolve the limitation of current methods and are especially suitable for the sparse mutation situation.

## RESULTS

### Framework of EMSI

**Figure 1.**
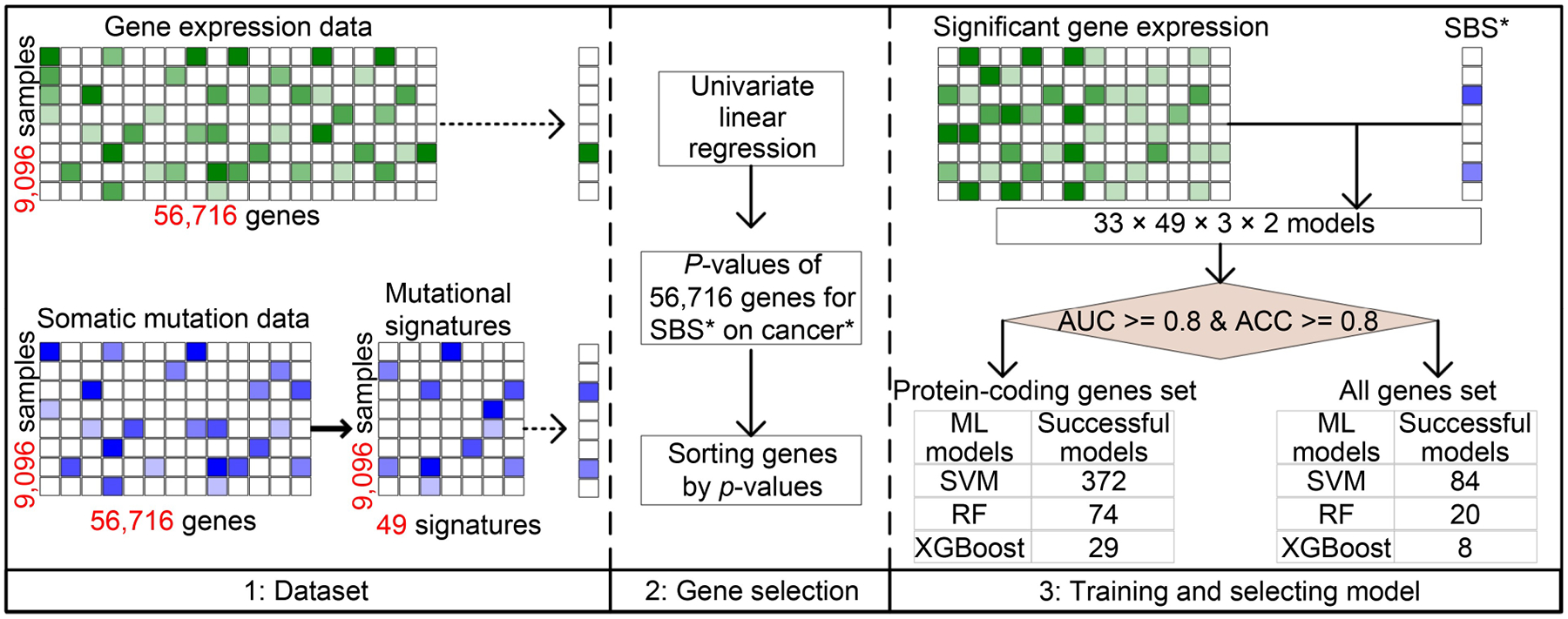
The flowchart of the study. In the Dataset section (left, panel 1), quantitative contributions of mutational signatures were solved from somatic mutation data. Gene expression data of 33 cancer types were collected from TCGA. In the Gene selection section (middle, panel 2), 56,716 *p*-values assessing the association between each gene and each mutational signature were obtained by using a univariate linear regression model. *P*-values were sorted in ascending order. In the Training and selecting model section (right, panel 3), expression data of all top-ranking genes were used to train a machine learning classifier to predict the binary contribution state of a mutational signature, using one of three models (SVM, RF, and XGBoost) each time. Because the work involved 33 cancer types, 49 mutational signatures, three training models, and two modalities (all genes or protein-coding genes only), we built totally 9,702 (33×49×3×2) candidate models. Finally, a total of 587 well-performing models with both accuracy and AUC above 80% on five-fold cross-validation were reserved. The asterisk symbol (*) tagged with the words “SBS” and “cancer” designates one particular SBS or cancer out of the total panel.

The overall study design is depicted in Fig. 1. This figure shows the basic framework of the EMSI (gene expression and mutational signature inference) model and the model selection results.

### Mutational signature computation

**Figure 2.**
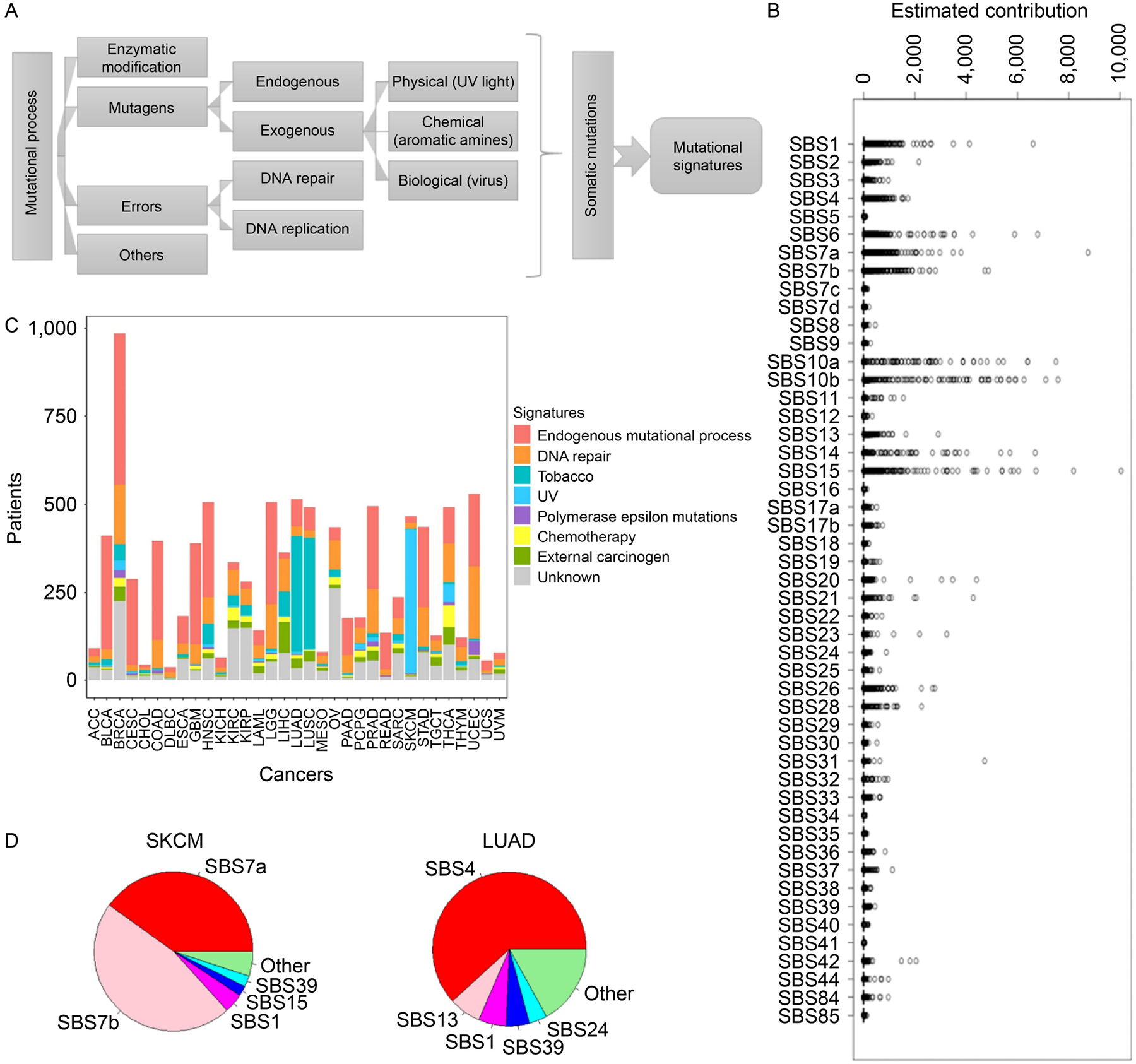
Mutational signatures resolved from TCGA somatic mutation data. (A) Various mutational processes lead to characteristic mutational signatures. (B) Quantitative contributions of each mutational signature to the 9,096 patients. “Contribution” is a quantitative value that assesses how much a particular mutational signature has contributed to the observed mutational catalog of a patient sample. (C) Dissection of each cancer type by category of contributing mutational signatures. A patient may exhibit contribution from multiple mutational signatures, but only the signature of the highest contribution was counted. (D) Mutational signature distribution in SKCM and LUAD. UV light damage signatures are the major signature in SKCM. A smoking-related signature is the major signature in LUAD.

A myriad of endogeneous/exogeneous mutational processes may modify the somatic genome, causing its deviation from the germline genome. The most characteristic somatic mutations are single base substitutions (SBS), and a specific combinatorial profile of diverse SBSs is conceptualized as a mutational signature that is associated with a particular mutational process (Fig. 2A). The mutational profiles of 9,096 cancer patients were decomposed to quantitative contributions from 49 the Catalogue Of Somatic Mutation In Cancer (COSMIC) mutational signatures. A signature’s contribution to a patient’s mutational profile is a non-negative value. Zero means the mutational signature does not contribute to that particular patient. A higher value denotes a relatively stronger contribution from a particular mutational signature. Each patient’s somatic mutations can be attributed to multiple mutational signatures. Overall contributions of the 49 COSMIC mutational signatures to all patients can be seen in Fig. 2B. For conciseness, we summarized the 49 mutational signatures into eight categories and dissected each cancer type by categories of contributing mutational signatures (Fig. 2C). Then, we investigated the composition of signature contribution for each cancer type, by considering the representative signature of each patient of that cancer type. For skin cutaneous melanoma (SKCM) and lung adenocarcinoma (LUAD), respectively, we sorted the mutational signatures decreasingly by their contribution and focused on the top five signatures with the highest contribution (Fig. 2D). The composition of the predominant signatures intuitively explains the major mutagens that are commonly associated with these two cancer types. In SKCM, the dominant mutational signatures are SBS7a and SBS7b; both are signatures resulting from UV light damage. In LUAD, the predominant signature is SBS4, which is the result of tobacco consumption. These results affirm the validity of our mutational signature calling from The Cancer Genome Atlas (TCGA) data.

To model the associations between mutational signature and gene expression, we used two gene pools: all genes and protein-coding genes. The gene pool of all genes has more power due to a wider selection of genes, but it has less universal applicability due to the higher possibility of missing genes during future applications. Conversely, the gene pool of exclusively protein-coding genes has less power due to a smaller selection of genes, but it has the advantage of more universal applicability. We performed machine learning in numerous scenarios each defined by a specific mutational signature and a specific cancer type. In each scenario, gene expression data were taken to characterize the patients of a cancer type, and three machine learning methods were alternatively utilized to classify the dichotomized contribution of a particular signature. A signature’s contribution to a patient was pre-dichotomized to 0 or 1, where 0 denotes the literal 0 value, and 1 denotes all non-zero values. During the model training, datasets with fewer than 50 patients in either signature positive or signature negative datasets were removed. In the end, out of a total of 1,617 (33×49) scenarios, 996 training attempts with five-fold cross-validation were conducted for each machine learning method using each gene pool.

### Models training with all genes

**Figure 3.**
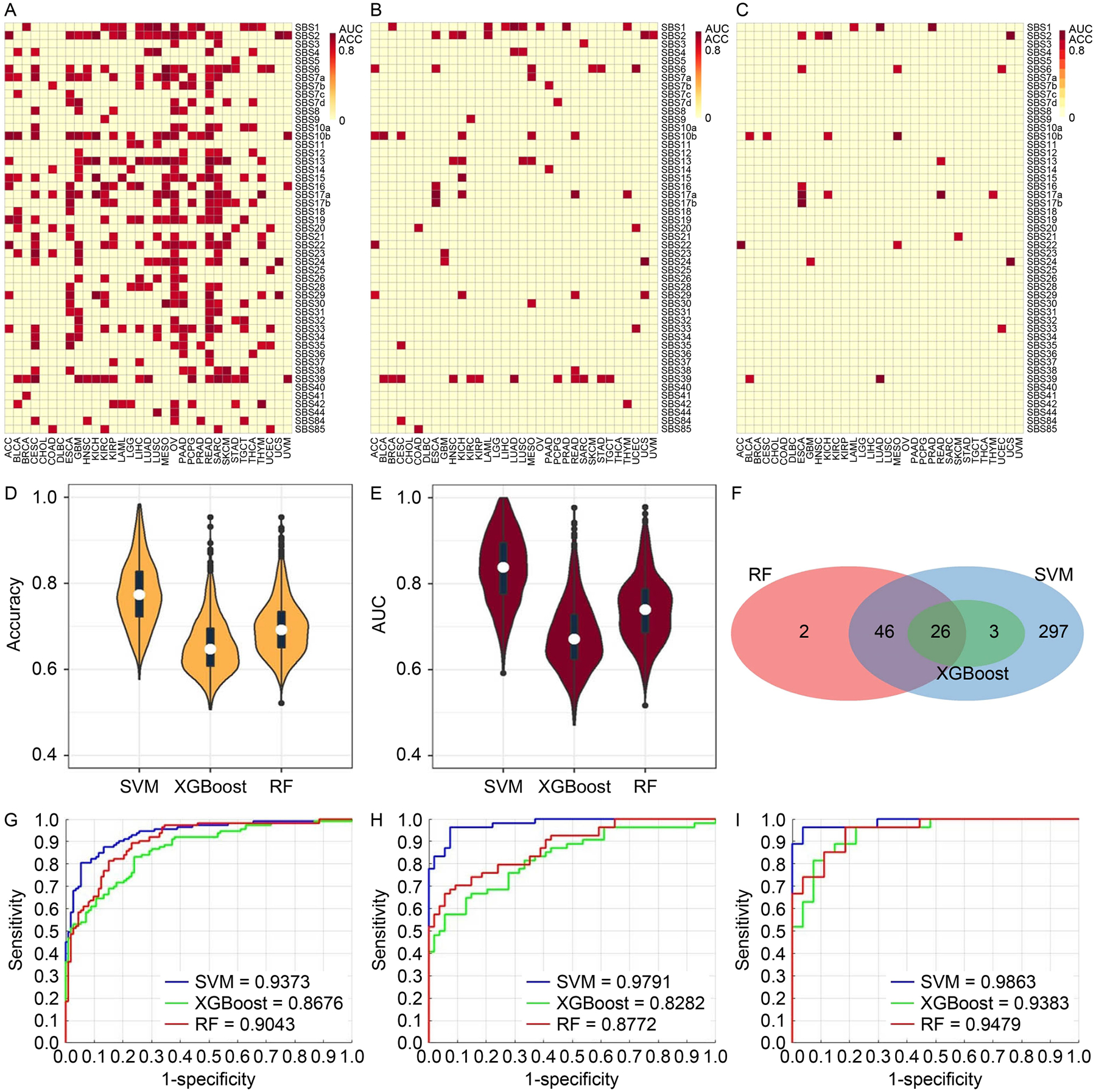
Results of classification models constructed using the all-gene pool. (A–C) Heatmaps and clusters of classification models using the average of accuracy and AUC from the three methods: SVM (A), RF (B), and XGBoost (C). Each cell in the heat map denotes a model (classification scenario) defined by a cancer type and a classification target, *i.e*., a mutational signature. A successful model is colored proportionally to the average of AUC and accuracy if both accuracy and AUC are above 0.8. Otherwise, the model is considered unsuccessful, and is represented with a uniform plain color. (D) Violin and boxplot of accuracy from the three methods. (E) Violin and boxplot of AUC from the three methods. (F) Venn diagram of the successful models among all three methods. One model is identified by the cancer type and the mutational signature. (G) Receiver-operating-characteristic curve (ROC) of model for SBS4 (smoking signature) in cancer type LUAD. (H) ROC of model for SBS4 (smoking signature) in cancer type LUSC. (I) ROC of model for SBS2 (AID/APOBEC signature) in cancer type UCS.

Three methods, Random Forest (RF), eXtreme Gradient Boosting (XGBoost), and Support Vector Machine (SVM) were used to model the associations between mutational signature and gene expression. We consider a model successful if both accuracy and AUC are greater than 80%. For the all-gene pool, 374 of the 996 classification scenarios were successfully resolved by at least one of the three methods. SVM performed the best with 372 successful models (Fig. 3A), and RF came second with 74 successful models (Fig. 3B), and XGBoost ranked bottom with 29 successful models (Fig. 3C). With the most successful model SVM, the number of successful models was highly uneven across cancer types. Some cancer types such as ovarian cancer (OV), kidney renal clear cell carcinoma (KIRC), and kidney renal papillary cell carcinoma (KIRP) had over 40 mutational signatures successfully modeled by gene expression. In contrast, cancer types such as uterine carcinosarcoma (UCS) and uveal melanoma (UVM) had less than five mutational signatures successfully modeled by gene expression. The discrepancy among the cancer types is a reflection of the heterogeneity within cancers, where cancer types vary by mutation and gene expression spectrum. The disparity in performance among the three methods is also reflected in the accuracy (Fig. 3D) and AUC (Fig. 3E) measurements where SVM took the commanding lead. Twenty-six models were successful for all three methods (Fig. 3F). For concrete examples of modeling excellence, we present receiver operating characteristics (ROC) plots of three high performance models: SBS4 (smoking signature) in the two types of lung cancers, lung adenocarcinoma (LUAD) (Fig. 3G) and lung squamous cell carcinoma (LUSC) (Fig. 3H), and SBS2 (AID/APOBEC signature) in uterine carcinosarcoma (UCS) (Fig. 3I). These classification scenarios, using the pool of all genes, achieved > 0.94 AUC when modeled by SVM.

### Model training with protein-coding genes

**Figure 4.**
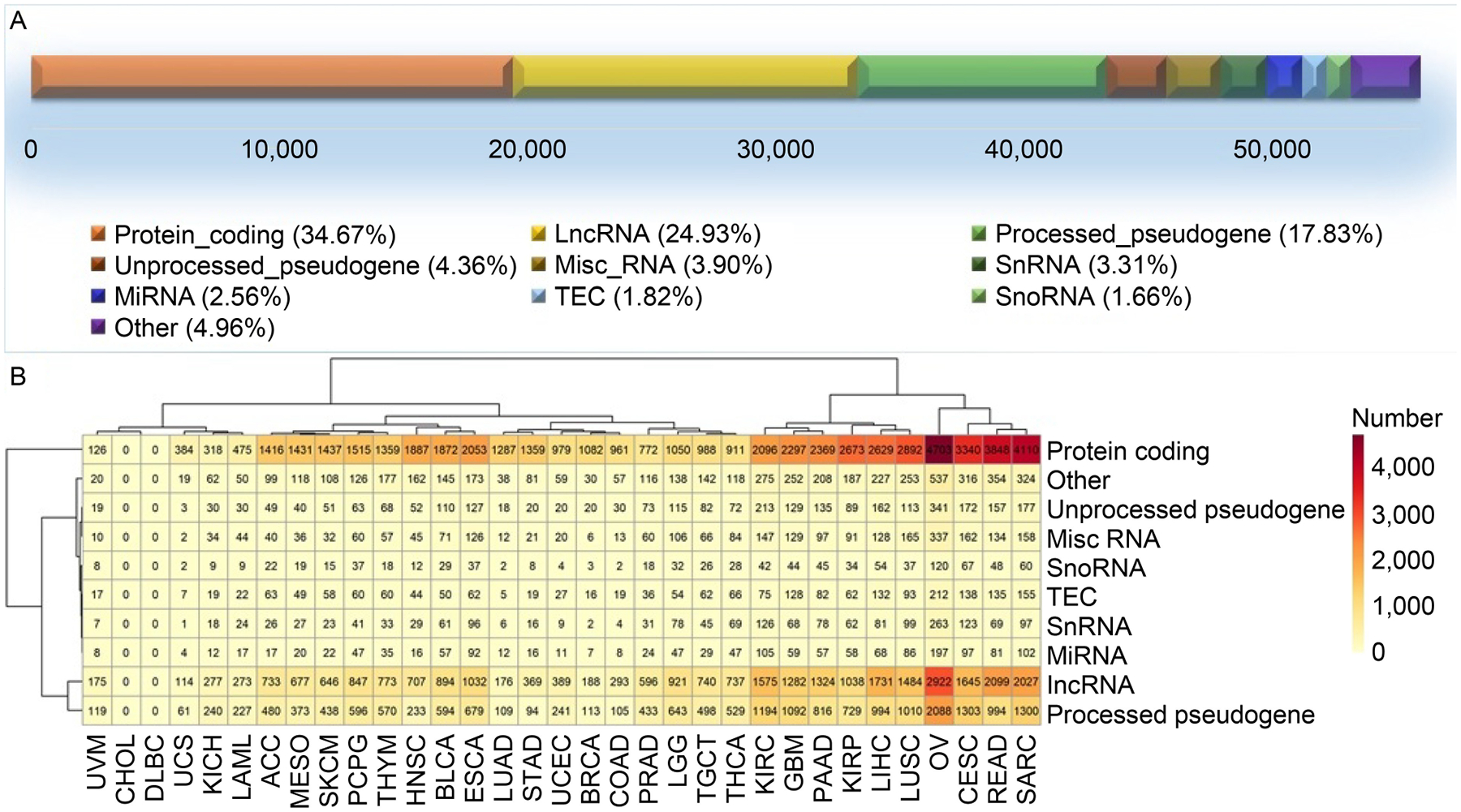
Gene type composition of the features recruited by SVM models where the pool of all genes was used. (A) The overall gene type composition from TCGA data. (B) Detailed gene type composition from all successful SVM models, stratified by cancer type in columns.

Of all 56,716 genes involved in the TCGA expression dataset, 34.7% represent protein-coding genes and the rest fall into various non-coding RNA categories (Fig. 4A). We examined gene type composition of the features recruited by SVM, the most successful model of the three. It was found that a large amount of non-coding RNAs are utilized in the successful models. In many cases, the proportions of non-coding RNAs are higher than protein-coding RNAs (Fig. 4B). LncRNA and pseudogenes were the two most predominant types of non-coding RNAs recruited by SVM models. This result strongly suggests that somatic mutations substantially affect non-coding RNA gene expression.

**Figure 5.**
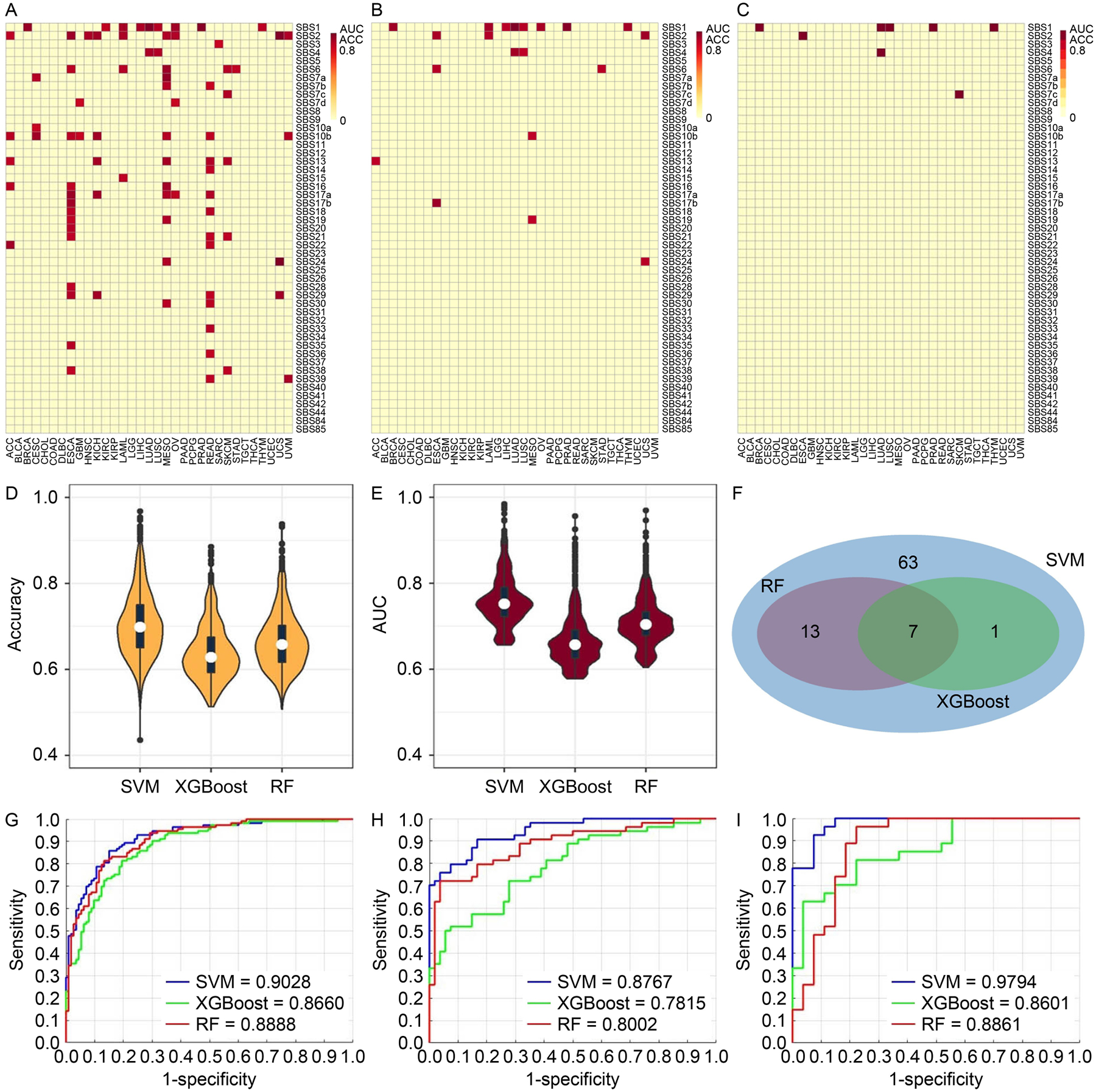
Results of classification models constructed using the pool of protein-coding-genes only. (A–C) Heatmaps and clusters of classification models using the average of accuracy and AUC from the three methods: SVM (A), RF (B), XGBoost (C). Graphic manifestation of these figures follows the same style as in Fig. 3. (D) Violin and boxplot of accuracy from the three methods. (E) Violin and boxplot of AUC from the three methods. (F) Venn diagram of the successful models among all three methods. (G) ROC of model for SBS4 (smoking signature) in cancer type LUAD. (H) ROC of model for SBS4 (smoking signature) in cancer type LUSC. (I) ROC of model for SBS2 (AID/APOBEC signature) in cancer type UCS.

When constrained to protein-coding genes, only 84 of all 996 training attempts passed our success criteria. Again, SVM performed the best with 84 successful models (Fig. 5A), RF came second with 20 successful models (Fig. 5B), and XGBoost still ranked bottom with 8 successful models (Fig. 5C). SVM models also produced the highest accuracy (Fig. 5D) and AUC (Fig. 5E). Seven were successful with respect to all three methods (Fig. 5F). Compare to the 26 models identified by all three methods using all genes, three models were shared between all genes and protein-coding genes (Supplementary Fig. S1). ROC plots of the same three high performance models as above (Fig. 3G–I) were plotted as excellent examples using the pool of protein-coding genes only (Fig. 5G–I). For these three classification scenarios, the constraint to protein-coding genes caused a unanimous drop in AUC as compared to using the all-gene pool. It is evident that non-coding genes are indispensable features to boost the mutational signature classification.

### Validations

**Figure 6.**
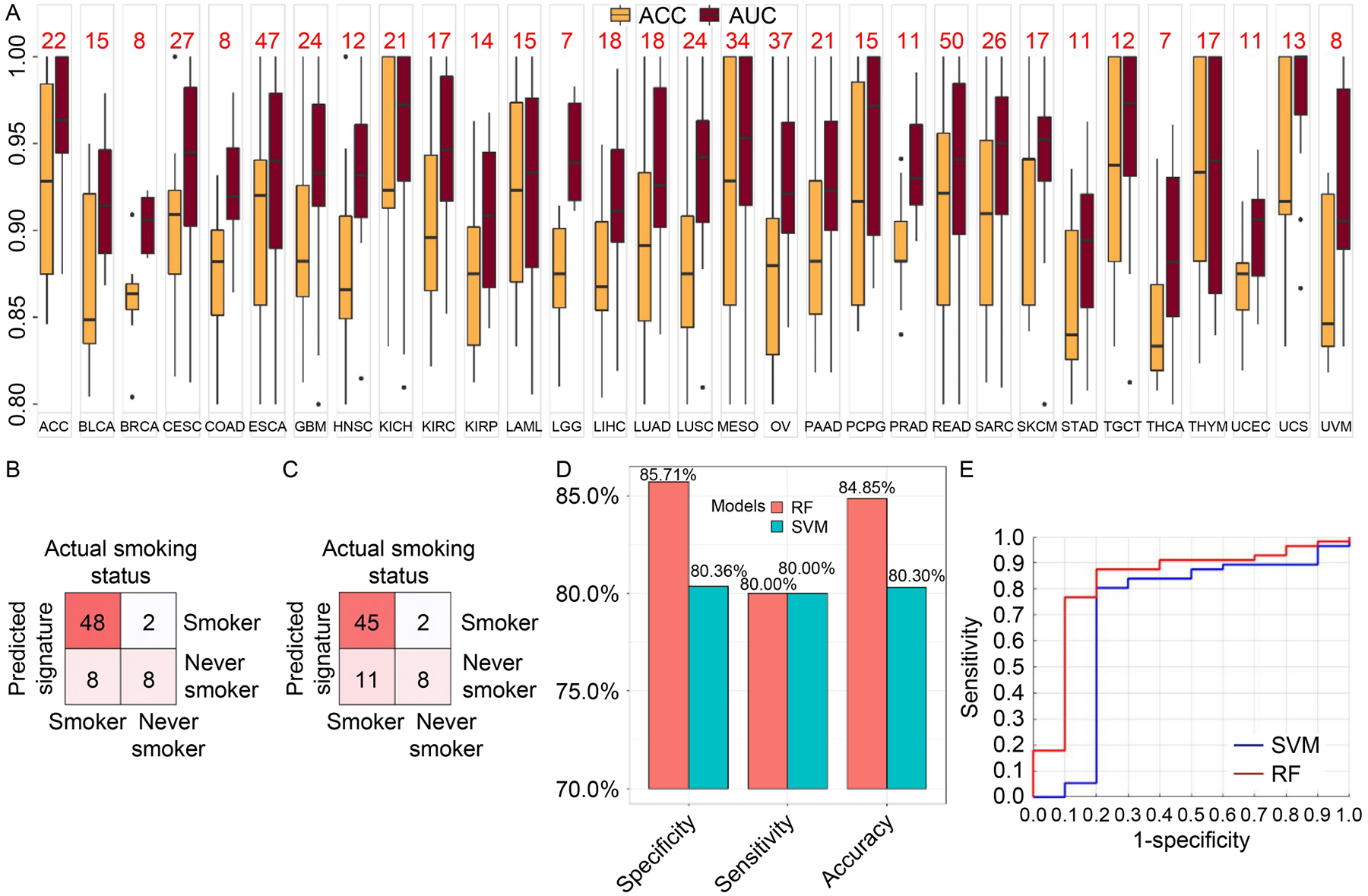
Validation results on two validation strategies. (A) Validation ACCs and AUCs of the 587 models selected from training. The red numbers on top of the boxplots represent the number of models for specific cancer. (B) Validation confusion table from RF model on GEO dataset GSE29016. (C) Validation confusion table results from SVM model on GSE29016. (D) Accuracy, specificity, and sensitivity from both SVM and RF models from GSE29016. (E) ROC produced by the SVM model and the RF model on GSE29016.

Validation was performed for the 587 models covering 31 cancer types selected from training on the 20% of TCGA data we initially left out. Accuracies and AUCs of all 587 models performed greater than 0.8 (Fig. 6A). An independent lung cancer expression dataset with smoking status was leveraged to validate the RF and SVM models on SBS4 classification trained on TCGA LUSC data. Because SBS4 has been clearly associated with tobacco intake, we designate the smoking status as a proxy for SBS4 labels. The patients involved in the external dataset were divided into 56 smokers and 10 never smokers, and thus we separated the dataset to SBS4-positive and SBS4-negative, accordingly. The overall confusion table of the validation results is displayed in Fig. 6B and Fig. 6C for RF and SVM, respectively. RF returned validation accuracy 84.8%, sensitivity 80.0%, and specificity 85.7%. SVM returned validation accuracy 80.3%, sensitivity 80.0%, and specificity 80.4% (Fig. 6D). The validation ROCs for RF and SVM are depicted in Fig. 6E.

Finally, some genes (including ZYG11A, RBM47, and KRT18) that are used in this model were regarded as an example to explore the biological implications. ZYG11A is an oncogene in non-small cell lung cancer (NSCLC) and it influences CCNE1 expression [15]. RBM47 is an RNA-binding protein and it inhibits NSCLC carcinoma metastasis [16]. Zhang *et al*. [17] found that EGR1 decreases the malignancy of human non-small cell lung carcinoma by regulating KRT18 expression. Since LUSC is a common pathological type of NSCLC, these genes are both associated with SBS4 and LUSC.

### Tumor heterogeneity and purity tests

**Figure 7.**
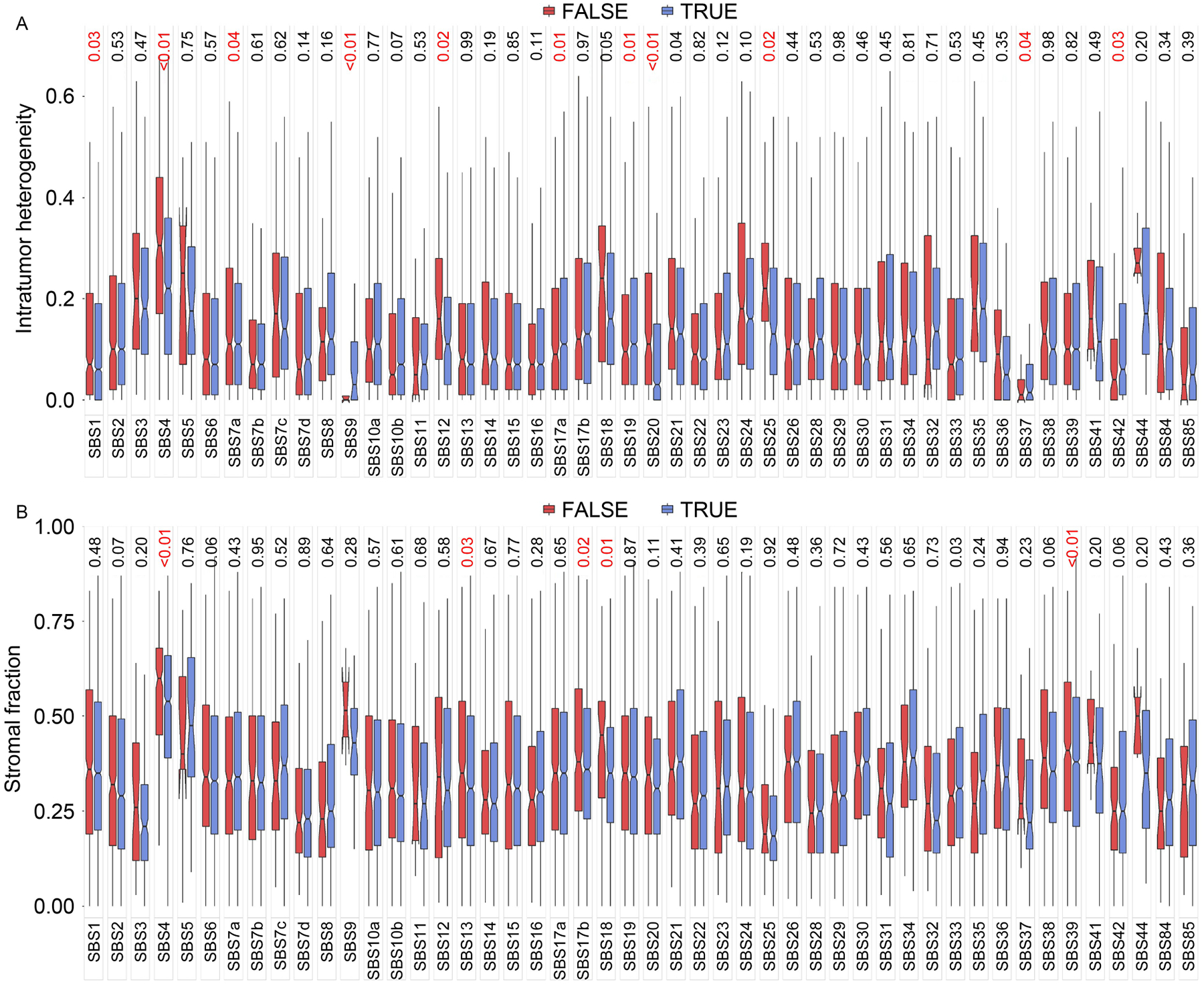
Heterogeneous tumors and tumor purity analysis against inferred results of 587 models. In total, 48 mutational signatures were covered. Values at the top of the image represent the *p*-values to assess the effect of heterogeneous tumors and tumor purity for the performance of models. The TRUE group comprises the samples that were correctly inferred and the FALSE group includes the samples that were wrongly inferred. (A) Results of heterogeneous tumors analysis. (B) Results of tumor purity analysis.

Tumors are highly heterogeneous and tumor samples often consist of tumor cells and stroma. We hypothesized that tumor heterogeneity and purity might be associated with model performance. To test these, we split the TCGA sample into correctly inferred (TRUE) with our models and incorrectly inferred (FALSE) with our models and compared tumor heterogeneity score (Fig. 7A) and stromal fraction (Fig. 7B) between TRUE and FALSE groups using *t*-test. For intratumor heterogeneity, 11 mutational signatures were significant with six mutational signatures (SBS1, SBS4, SBS7a, SBS12, SBS20, and SBS25) show higher intratumor heterogeneity in the FALSE group and five mutational signatures (SBS9, SBS17a, SBS19, SBS37, and SBS42) show higher intratumor heterogeneity in the TRUE group. For stromal fraction, six mutational signatures were significant with five mutational signatures (SBS4, SBS13, SBS17a, SBS18, and SBS39) show higher stromal fraction in the FALSE group and one mutational signature (SBS33) show higher stromal fraction in the TRUE group.

Finally, in a combination of PHP, Javascript, and R, we assembled all successful models to form an online form (EMSI) as a component of the MutEx analysis suite [14]. Models can be used or downloaded at the website of innovebioinfo.

## DISCUSSION

The field of mutational signatures made great strides over the past seven years, yet the full potential of this approach has not been realized. Mutational signatures analysis provides a vessel for identifying unique patterns of mutations that are characteristic of a specific mutagen. In principle, there are three main avenues for leveraging mutational signatures in biomedical research and applications. First, in cancer epidemiology, mutational signatures allow quantifying exposures of known carcinogens or identifying previously unknown carcinogens. For example, mutational signatures analysis was able to pinpoint azathioprine as the cause of cancer in transplant patients [18]. In another example, mutational signatures analysis was used to quantify the abundance of aristolochic acid in liver cancer in Asia; the analysis revealed that 78% of liver cancer in Taiwan, China is caused by aristolochic acid, thus illuminating an unexpected path for cancer prevention [19]. Second, in cancer treatment, mutational signatures can be used as a biomarker for a failure of different DNA repair pathways. Previous research has shown that many human cancers exhibit BRCA-like mutational signature without harboring any *BRCA1*, *BRCA2*, or *PALB2* mutations [20,21]. This has provided an opportunity to target existing treatments (*i.e*., mainly PARP inhibitors), and initial results indicate that patients respond to these treatments [22,23]. Third, in basic DNA repair and mutagenesis research, mutational signatures analysis allows delineating the source of individual somatic mutations. More specifically, we can now probabilistically attribute a mutational signature to each somatic mutation in a cancer genome. This allows a better understanding of the genomic and epigenomic modifiers of mutagenesis and carcinogenesis. For example, it has been demonstrated that replication timing and nucleo-some occupancy affect only certain mutational processes (*e.g*., ones due to the APOBEC family of deaminases) [24]. Others have used mutational signatures to describe the interaction between DNA repair machinery and DNA replication polymerases [25]. Overall, a mutational signature is a powerful tool that can be leveraged in cancer epidemiology, cancer treatment, and basic scientific research.

Gene expression dysregulation is a major research topic in cancer research. Thousands of cancer gene expression studies have been conducted to yield myriads of gene expression data that are archived in public repositories. These data provide a unique opportunity to study the underlying mutational signatures that played a part in regulating the expression profiles. In this study, we leveraged both somatic mutation and gene expression data from 33 cancer types to model the association between mutational signature and gene expression. Of the three methods we tried, SVM achieved the highest AUC. The other two methods RF and XGBoost had a moderate performance. On average, around 65% of known mutational signatures can be modeled by gene expression per cancer type. This indicates that the regulatory effect between somatic mutation and gene expression alteration is not ubiquitous. Not every somatic mutation can exert an effect on gene expression. This observation is consistent with the eQTL analysis results where only a subset of SNPs are found to regulate gene expression [26]. Thus, we limited our models to those with accuracy and AUC greater than 80% for future inference applications. Our study suggests that somatic mutation can also dysregulate non-coding RNA expression. By encompassing non-coding RNAs, the models built generally increased overall fitness substantially. This result is consistent with a previous finding that cancer prognosis can be improved by the combination of protein-coding and non-coding RNAs [27]. Variation in the performance of these three methods may be dependent on scenarios. For example, XGBoost has been found to perform better with a larger sample size [28], SVM has been found to work better with a smaller sample size [29,30].

Our study is also subjected to several limitations. Mutational signature analysis is commonly based on non-negative matrix factorization, an unsupervised process. The results were then assigned to known cancer etiology. This approach causes many of the mutational signatures to be of unknown cancer etiology. These inherent limitations of mutational signatures are beyond our control in this study. Furthermore in this study, we focused on single base substitution mutational signatures and omitted doublet base substitution signatures and small insertion and deletion signatures, which are relatively harder to model. Besides, in an ideal scenario, the best validation is by an independent dataset. However, proper validation datasets require paired somatic mutation and gene expression data, which are exceptionally rare. Thus, we used an existing lung squamous carcinoma gene expression dataset comparing smokers and non-smokers to validate the smoking-related mutational signature, and achieved respectable validation accuracy. There are several key points to take note regarding this validation attempt. First, whereas the models were all trained based on total RNA-seq data, the validation data was microarray-based. In addition to the difference in transcriptome profiling technology, the microarray is also characteristic in that the dataset does not contain non-coding RNAs. Thus, models using protein-coding genes that have less inference power were chosen for validations. We expect stronger validation performance from total RNA-seq data. Even though this validation was applied to only one of the 49 mutational signatures in one cancer type, it proves that the concept can be generalized toward other mutational signatures. Our study reveals the key message that some somatic mutations can lead to gene expression alteration consequences. By modeling these associations between mutational signature and gene expression, we can potentially infer mutational signatures in future RNA-seq studies.

## MATERIALS AND METHODS

### Data acquisition

Gene expression data (RNA-seq FPKM) of 10,274 cancer patients and somatic mutation data of 10,179 patients (reference genome GRCh38) from 33 cancer types were downloaded from Genomic Data Commons, the gateway of The Cancer Genome Atlas (TCGA). The gene expression data contains 56,716 genes which include both protein-coding and non-coding genes. Mutual integration of the two data sources retained 9,096 common patients. The cancer type abbreviations, full name, and detailed sample size are available in Supplementary Table S1. Probability matrix for 49 established COSMIC reference mutational signatures (v3) was downloaded from Synapse Documentation at synapse website (Synapse:syn11738319) (Supplementary Table S2). As we briefly described in the Introduction, we formalized a catalog of 96 three-nucleotide motifs that surround the mutational focus (one upstream nucleotide + mutation site + one downstream nucleotide site) and derived frequency tables of this motif catalog for each involved patient. We leveraged a computational function from R package MutationalPatterns [31] to fit the patient mutational motif frequency tables to the reference mutational signatures while requiring the coefficients, *i.e*., signature-to-patient contribution strengths, be non-negative values. The estimated coefficients came out in the form of a 96-by-9,096 matrix of non-negative values. During model training, the mutational signature for each sample is dichotomized into 0 (signature-to-patient contribution equals 0) and 1 (signature-to-patient contribution greater than 0) where 0 denote that this mutational signature is not present in this sample, and 1 denotes this mutational signature is present in this sample.

### Study design

Our work entailed a gene expression dataset that involves 56,716 genes. A majority (65.3%) of the genes are non-coding genes. Univariate linear regression was first performed to assess associations between one gene and one mutational signature within a cancer type (R function GLM, mutational signature ~ gene, family = gaussian). This is repeated for all genes, all mutational signature and all cancer types. In succession to the univariate linear regression, three machine learning methods were used to model the associations between mutational signature and gene expression. The three methods were RF as conveyed through R package randomForest, XGBoost through R package (xgboost), and SVM through R package e1071. Both RF and XGBoost are decision tree-based methods; the major difference is that RF builds all trees simultaneously while XGBoost builds one tree at a time and tries to correct errors from the previous tree. SVM is a different type of classification and regression analysis that is based on separation by hyperplanes. These three methods are supervised machine learning methods with strengths and weaknesses. The TCGA data were split into 80% training and 20% validation. Five fold cross-validations were applied on all model training.

For a specific mutational signature within a cancer type, genes were ranked by significance based on *p*-value from univariate linear regression. At *p* < 0.05, no more than 600 differentially expressed genes were identified for a mutational signature in a cancer type. To determine the optimal number of genes for each model, we used an iterative approach. Each model was assessed with 10 to 600 top-ranked genes with an increment of 10 genes in each iteration. And models with the best performing AUC were selected. Model fittings were performed twice, once for all genes which include protein-coding and non-protein-coding genes, and once for protein-coding genes. Based on the strategy of selecting the optimal models, different models may have different numbers of optimal genes. In addition to the 20% of the TCGA dataset used as validation, an independent gene expression dataset on lung cancer patients with known smoking status was downloaded from gene expression omnibus (GEO accession No. GSE29016) to validate the inference accuracy of the smoking-related mutational signature.

Association between the performance of machine learning models and two tumor factors (stromal fraction and intratumor heterogeneity) were discussed based on 587 models covering 48 mutational signatures. Stromal fraction and intratumor heterogeneity values for TCGA samples were obtained from the study [32] by Thorsson *et al*. All inferred labels of 587 models on five-fold cross-validations were split into two categories including TRUE and FALSE where TRUE represents the samples that were correctly inferred and the FALSE represents the samples that were wrongly inferred. For a specific signature, a *t*-test was used to assess if there is a significant difference between TRUE and FALSE groups for stromal fraction and intratumor heterogeneity for all models.

## Supplementary Material

supplementary 2

supplementary 1

## ABBREVIATIONS

AESA: Advanced expression survival analysis
ACC: Adrenocortical carcinoma
BLCA: Bladder urothelial carcinoma
BRCA: Breast invasive carcinoma
CESC: Cervical squamous cell carcinoma and endocervical adenocarcinoma
CHOL: Cholangiocarcinoma
COAD: Colon adenocarcinoma
DLBC: Lymphoid neoplasm diffuse large B-cell lymphoma
eQTL: gene expression quantitative trait loci
ESCA: Esophageal carcinoma
GBM: Glioblastoma multiforme
HNSC: Head and neck squamous cell carcinoma
KICH: Kidney chromophobe
KIRC: Kidney renal clear cell carcinoma
KIRP: Kidney renal papillary cell carcinoma
LAML: Acute myeloid leukemia
LGG: Brain lower grade glioma
LIHC: Liver hepatocellular carcinoma
LUAD: Lung adenocarcinoma
LUSC: Lung squamous cell carcinoma
MESO: Mesothelioma
OV: Ovarian serous cystadenocarcinoma
PAAD: Pancreatic adenocarcinoma
PCPG: Pheochromocytoma and paraganglioma
PRAD: Prostate adenocarcinoma
READ: Rectum adenocarcinoma
RF: Random forest
ROC: Receiver operating characteristics
SARC: Sarcoma
SBS: Single base substitutions
SKCM: Skin cutaneous melanoma
STAD: Stomach adenocarcinoma
SVM: Support vector machine
TCGA: The Cancer Genome Atlas
TGCT: Testicular germ cell tumors
THCA: Thyroid carcinoma
THYM: Thymoma
UCEC: Uterine corpus endometrial carcinoma
UCS: Uterine carcinosarcoma
UV: Ultraviolet
UVM: Uveal melanoma
XGBoost: EXtreme Gradient Boosting
