# Supplementary material for: Modeling the relationship between gene expression and mutational signature
Source: Quant Biol. 2023 Mar 1;11(1):31–43. doi: 10.15302/J-QB-022-0309 (PMC10078980; doi:10.15302/J-QB-022-0309)

Supplemental materials

Supplementary Table 1. Cancer names, abbreviations, and sample size.

Supplementary Table 2. Mutational signature ID and etiology.

Supplementary Figure 1. Venn diagram showing common models by all genes and protein-coding genes.

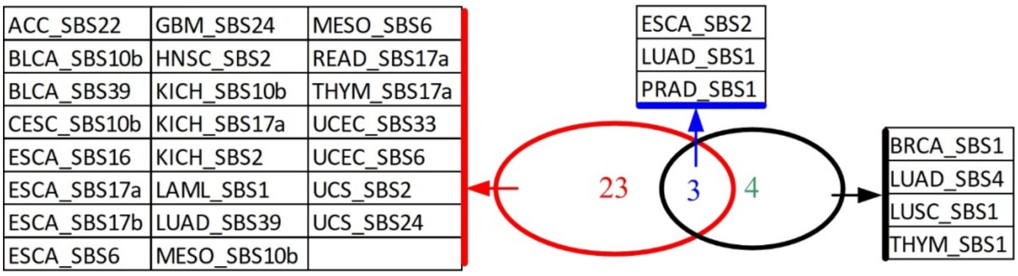

Supplement: Supplementary file 1 — Supplementary Information [file QUB2-11-31-s001.pdf]
