# Supplementary material for: Modeling the relationship between gene expression and mutational signature
Source: Quant Biol. 2023 Mar 1;11(1):31–43. doi: 10.15302/J-QB-022-0309 (PMC10078980; doi:10.15302/J-QB-022-0309)
Supplement: Supplementary file 2 — Supplementary Information [file QUB2-11-31-s002.pdf]

Supplementary Table 1. Cancer names, abbreviation and sample size

Cancer Abbreviation	Cancer Full Name	Sample Size
ACC	Adrenocortical Carcinoma	79
BLCA	Bladder Urothelial Carcinoma	408
BRCA	Breast Invasive Carcinoma	979
CESC	Cervical Squamous Cell Carcinoma and Endocervical Adenocarcinoma	286
CHOL	Cholangiocarcinoma	36
COAD	Colon Adenocarcinoma	394
DLBC	Lymphoid Neoplasm Diffuse Large B-cell Lymphoma	37
ESCA	Esophageal Carcinoma	160
GBM	Glioblastoma Multiforme	149
HNSC	Head and Neck Squamous Cell Carcinoma	493
KICH	Kidney Chromophobe	65
KIRC	Kidney Renal Clear Cell Carcinoma	332
KIRP	Kidney Renal Papillary Cell Carcinoma	278
LAML	Acute Myeloid Leukemia	112
LGG	Brain Lower Grade Glioma	502
LIHC	Liver Hepatocellular Carcinoma	359
LUAD	Lung Adenocarcinoma	509
LUSC	Lung Squamous Cell Carcinoma	489
MESO	Mesothelioma	81
OV	Ovarian Serous Cystadenocarcinoma	272
PAAD	Pancreatic Adenocarcinoma	169
PCPG	Pheochromocytoma and Paraganglioma	178
PRAD	Prostate Adenocarcinoma	492
READ	Rectum Adenocarcinoma	132
SARC	Sarcoma	235
SKCM	Skin Cutaneous Melanoma	103
STAD	Stomach Adenocarcinoma	372
TGCT	Testicular Germ Cell Tumors	128
THCA	Thyroid Carcinoma	487
THYM	Thymoma	118
UCEC	Uterine Corpus Endometrial Carcinoma	526
UCS	Uterine Carcinosarcoma	56
UVM	Uveal Melanoma	80

Supplementary Table 2. Mutational signature ID and etiology

Signature	Etiology
**SBS1**	An endogenous mutational process initiated by spontaneous or enzymatic deamination of 5-methylcytosine to thymine which generates G:T mismatches in double stranded DNA. Failure to detect and remove these mismatches prior to DNA replication results in fixation of the T substitution for C.
**SBS2**	Attributed to activity of the AID/APOBEC family of cytidine deaminases on the basis of similarities in the sequence context of cytosine mutations caused by APOBEC enzymes in experimental systems. APOBEC3A is probably responsible for most mutations in human cancer, although APOBEC3B may also contribute (these differ in the sequence context two bases 5' to the mutated cytosine, see 1,536 mutation classification signature extraction). SBS2 mutations may be generated directly by DNA replication across uracil or by error prone polymerases replicating across abasic sites generated by base excision repair removal of uracil.
**SBS3**	Defective homologous recombination-based DNA damage repair which manifests predominantly as small indels and genome rearrangements due to abnormal double strand break repair but also in the form of this base substitution signature.
**SBS4**	Associated with tobacco smoking. Its profile is similar to the mutational spectrum observed in experimental systems exposed to tobacco carcinogens such as benzo[a]pyrene. SBS4 is, therefore, likely due to direct DNA damage by tobacco smoke mutagens.
**SBS5**	**Unknown** SBS5 mutational burden is increased in bladder cancer samples with ERCC2 mutations and in many cancer types due to tobacco smoking.
**SBS6**	SBS6 is associated with defective DNA mismatch repair and is found in microsatellite unstable tumours.
**SBS7a**	SBS7a/SBS7b/SBS7c/SBS7d are found in cancers of the skin from sun exposed areas and are thus likely to be due to exposure to ultraviolet light. SBS7a may possibly be the consequence of just one of the two major known UV photoproducts, cyclobutane pyrimidine dimers or 6-4 photoproducts. However, there is currently no evidence for this hypothesis and it is unclear which of these photoproducts may be responsible for SBS7a.
**SBS7b**	SBS7a/SBS7b/SBS7c/SBS7d are found in cancers of the skin from sun exposed areas and are likely to be due to exposure to ultraviolet light. SBS7b may possibly be the consequence of just one of the two major known UV photoproducts, cyclobutane pyrimidine dimers or 6-4 photoproducts. However, there is no evidence for this hypothesis and it is unclear which of these photoproducts may be responsible for SBS7b.
**SBS7c**	SBS7a/SBS7b/SBS7c/SBS7d are found in cancers of the skin from sun exposed areas and are likely to be due to exposure to ultraviolet light. SBS7c is possibly the consequence of translesion DNA synthesis by enzymes with propensity to insert T, rather than A, opposite ultraviolet induced thymidine and cytidine photodimers. The preponderance of T>A rather than T>C mutations may reflect the heavier burden of thymidine compared to cytidine dimers induced by UV light.
**SBS7d**	SBS7a/SBS7b/SBS7c/SBS7d are found in cancers of the skin from sun exposed areas and are likely to be due to exposure to ultraviolet light. SBS7d is possibly the consequence of translesion DNA synthesis by error-prone polymerases with greater

	propensity to insert G, rather than A, opposite UV light induced thymidine and cytidine photodimers.
**SBS8**	**Unknown**
**SBS9**	May be due in part to mutations induced during replication by polymerase eta as part of somatic hypermutation in lymphoid cells.
**SBS10a**	Polymerase epsilon exonuclease domain mutations.
**SBS10b**	Polymerase epsilon exonuclease domain mutations.
**SBS11**	SBS11 exhibits a mutational pattern resembling that of alkylating agents. Patient histories indicate an association between previous treatment with the alkylating agent temozolomide and SBS11 mutations.
**SBS12**	**Unknown**
**SBS13**	Attributed to activity of the AID/APOBEC family of cytidine deaminases on the basis of similarities in the sequence context of cytosine mutations caused by APOBEC enzymes in experimental systems. APOBEC3A is probably responsible for most mutations in human cancer, although APOBEC3B may also contribute (these differ in the sequence context two bases 5’ to the mutated cytosine, see 1536 mutation classification signature extraction). SBS13 mutations are likely generated by error prone polymerases (such as REV1) replicating across abasic sites generated by base excision repair removal of uracil.
**SBS14**	Concurrent polymerase epsilon mutation and defective DNA mismatch repair.
**SBS15**	Defective DNA mismatch repair.
**SBS16**	**Unknown**
**SBS17a**	**Unknown**
**SBS17b**	**Unknown**
**SBS18**	Possibly damage by reactive oxygen species.
**SBS19**	**Unknown**
**SBS20**	Concurrent POLD1 mutations and defective DNA mismatch repair.
**SBS21**	DNA mismatch repair deficiency.
**SBS22**	Aristolochic acid exposure. Found in cancer samples with known exposures to aristolochic acid and the pattern of mutations exhibited by the signature is consistent with that observed in experimental systems of aristolochic acid exposure.
**SBS23**	**Unknown**
**SBS24**	Aflatoxin exposure. SBS24 has been found in cancer samples with known exposures to aflatoxin and the pattern of mutations exhibited by the signature is consistent with that observed in experimental systems exposed to aflatoxin.
**SBS25**	**Unknown** However, some Hodgkin’s cell line samples in which the signature has been found were from patients exposed to chemotherapy and it is possible that SBS25 is due to chemotherapy treatment.
**SBS26**	Defective DNA mismatch repair.
**SBS28**	**Unknown**
**SBS29**	SBS29 has been found in cancer samples from individuals with a tobacco chewing habit.
**SBS30**	SBS30 is due to deficiency in base excision repair due to inactivating mutations in NTHL1.
**SBS31**	Prior chemotherapy treatment with platinum drugs.
**SBS32**	Prior treatment with azathioprine to induce immunosuppression. Associated mutation classes and signatures

**SBS33**	**N/A**
**SBS34**	**Unknown**
**SBS35**	Prior chemotherapy treatment with platinum drugs.
**SBS36**	Defective base excision repair, including DNA damage due to reactive oxygen species, due to biallelic germline or somatic MUTYH mutations.
**SBS37**	**Unknown**
**SBS38**	**Unknown** Found only in ultraviolet light associated melanomas suggesting potential indirect damage from UV-light.
**SBS39**	**Unknown**
**SBS40**	**Unknown**
**SBS41**	**Unknown**
**SBS42**	Occupational exposure to haloalkanes.
**SBS44**	Defective DNA mismatch repair.
**SBS84**	Activity of activation-induced cytidine deaminase (AID).
**SBS85**	Indirect effects of activation-induced cytidine deaminase (AID) induced somatic mutagenesis in lymphoid cells.
